# The antitumor efficiency of combined electrochemotherapy and single dose irradiation on a breast cancer tumor model

**DOI:** 10.2478/v10019-012-0035-x

**Published:** 2012-06-19

**Authors:** Elham Raeisi, Seyed Mahmoud Reza Aghamiri, Azin Bandi, Negar Rahmatpour, Seyed Mohammad Firoozabadi, Sedigheh Amini Kafi-Abad, Lluis M Mir

**Affiliations:** 1 Department of Medical Physics, Faculty of Medical Science, Tarbiat Modares University, Tehran, Iran; 2 Faculty of Science, University of Geneve, Geneve, Switzerland; 3 Division of Radiation Medicine, Shahid Beheshti University, Tehran, Iran; 4 Department of Immunohematology, Research Center of Iranian Blood Transfusion Organization (IBTO), Tehran, Iran; 5 CNRS, Orsay, Laboratoire de Vectorologie et Therapeutiques Anticancereuses, UMR 8203, 91405 Orsay cedex, France; 6 Universite Paris-Sud, Laboratoire de Vectorologie et Therapeutiques Anticancereuses, UMR 8203, 91405 Orsay cedex, France; 7 Institute Gustave Roussy, Laboratoire de Vectorologie et Therapeutiques Anticancereuses, UMR 8203, 114 rue E. Vaillant, 94805 Villejuif cedex, France

**Keywords:** cisplatin, irradiation, electroporation, invasive ductal carcinoma, multimodalities

## Abstract

**Background:**

The aim of this study was to investigate the antitumor effectiveness of electrochemotherapy with cisplatin combined with suboptimal radiotherapy doses. Tumor radiosensitization was evaluated on large invasive ductal carcinoma tumors in Balb/C mice.

**Materials and methods:**

Tumors of an average volume of 630 mm^3^ were treated with cisplatin, electric pulses, radiotherapy, electrochemotherapy, alone as well as in appropriate combinations. Tumors were irradiated with Cobalt-60 γ-rays at doses 3 Gy and 5 Gy in combination with electrochemotherapy using cisplatin. Controls included each of the treatments alone as well as the combination of the radiotherapy with electric pulses alone or with cisplatin alone. Antitumor effectiveness was evaluated by tumor growth delay, tumor-doubling time, inhibition ratio and the objective response rates.

**Results:**

As anticipated, electrochemotherapy was more effective than the treatment with cisplatin alone or the application of the electric pulses alone. When treatments were combined with tumor irradiation at either 3 or 5 Gy, the combination with electrochemotherapy was more effective: at 5 Gy, 2 animals out of 8 were in complete remission 100 days later. In general the higher 5 Gy dose of γ-radiation was more effective than the lower one of 3 Gy.

**Conclusions:**

The results of our study demonstrate that irradiation doses, 3 Gy or 5 Gy, increase the antitumor effectiveness of electrochemotherapy with cisplatin on invasive ductal carcinoma tumors. Good antitumor results were achieved in experimental tumors with a size comparable to clinical lesions, demonstrating that this three-modality combined treatment is useful for the treatment of large lesions even at sub-optimal radiotherapy doses.

## Introduction

Cisplatin is an anti-cancer chemotherapeutic drug that is administered to treat a large number of cancers such as those of the ovary, head and neck, cervix and bladder, carcinomas as well as small-cell and non-small-cell lung cancers.^1^–^5^ However, resistance to cisplatin is a clinical problem for the treatment of some tumors.^6^ After an initial response to chemotherapy, tumors subsequently show the minimal responsiveness to cisplatin.^1^,^2^,^7^ Besides direct cytotoxic effects, cisplatin has also the potential to enhance radiation-induced cell killing.^8^–^10^ Cisplatin as a radiosensitizer increases the damage to the nuclear DNA of malignant cells, enhancing the anti-neoplastic efficacy of the radiotherapy.^9^,^10^ The combination of cisplatin and radiation is a common treatment modality with synergistic effects for cancers.^4^,^5^,^8^,^9^,^11^ Several *in vitro* and *in vivo* studies have shown higher response rates, prolonged mean survival, increased survival rates, longer local recurrence-free survival rates, and considerable organ preservation with the use of cisplatin in combination with external radiotherapy.^8^–^11^ Indeed, it has been proven that cisplatin enhances the cytotoxicity of radiation on cells *in vitro*, on experimental tumors *in vivo* and in solid tumors in the clinic.^12^–^14^

Many studies have been performed to potentiate cisplatin antitumor effectiveness. One way to increase the radiosensitizing effect of cisplatin can result from the increase of the cisplatin intracellular accumulation. Several studies have been conducted using different drug delivery systems to increase the amount of cisplatin in the tumor cells.^8^,^14^–^18^ Electropermeabilization is a physical method that uses short and intense electric pulses to facilitate drug delivery into the cells.^19^,^20^ Electrochemotherapy (ECT) combines the administration of specific chemotherapeutic drugs (bleomycin or cisplatin) with the application of electric pulses to the tumors to locally increase drug uptake and thus drug effectiveness.^17^,^21^ Electrochemotherapy with bleomycin and cisplatin was elaborated *in vitro*, *in vivo* and in clinical trials.^15^,^16^,^21^–^25^ Besides permeability, electroporation has other effects like a transient and reversible reduction of the blood flow.^26^ This reduction may contribute to the entrapment of the drug in the tumor, thereby providing more time for the drug to act. As this effect would reduce drug wash-out from the tumor, it could play a role in antitumor efficiency.

It has been demonstrated that the electroporation of tumors increases the radiosensitizing effect of cisplatin on small experimental tumors.^27^,^28^ The enhanced radiosensitizing effect of cisplatin is actually due to the increased electroporation-mediated cisplatin delivery into the tumor cells.^29^ These initial studies used an already efficient dose of radiotherapy (RT) which was delivered to SA-1 fibrosarcoma transplanted in mice, combined to electrochemotherapy using cisplatin as well as small tumors in mice.^27^

The aim of the present study was to evaluate the efficiency of electrochemotherapy with cisplatin combined with a single radiotherapy dose on an animal model tumor of breast adenocarcinoma to determine the potentialities of this combination. Two sub-optimal radiotherapy conditions, 3 Gy or 5 Gy, unable to generate long term partial responses in this tumor model, were applied in these combinations. Moreover, it was decided to treat very large experimental tumors, of an average volume of 630 mm^3^, to assess the efficacy of the treatment alone or in combination in a preclinical model exploring the possibility of efficiently treating large lesions.

## Materials and methods

### Mice and tumors

Female Balb/C mice, purchased from the Pasteur Institute (Tehran, Iran) were maintained at 25°C with natural day/night cycle for 10 days for adaptation. Mice were 6–8 weeks old at the beginning of the experiments. A fragment (4 mm^3^) of a spontaneous mouse mammary tumor (an invasive ductal carcinoma) was transplanted subcutaneously into the flank of each animal.^30^,^31^ Two weeks after the implantation, when the tumors reached approximately 630 mm^3^ in volume (12–15 mm in diameter), the mice were randomly divided into the experimental groups (8–10 animals for each control and each experimental group). The study was approved by the local Research and Medical Ethics Committee, in accordance with the Shahid Beheshti University of Medical Sciences Guidelines for the Care and Use of Laboratory Animals.

### Cisplatin

Cisplatin (50 mg/ml, Ebewe Pharma, Austria) was diluted with 0.9% NaCl the day of the treatment. Cisplatin at a dose of 8 mg/kg was injected intratumorally and the injected volume was adjusted to deliver 0.02 ml of this cisplatin solution per gram of body weight.

### Electrochemotherapy

Two flat stainless steel plates were mounted on a caliper to serve as 20 × 20 mm parallel electrodes. A distance between electrodes was adjusted by rolling of the caliper depending on the tumor size. The voltage value was then set in an ECT-SBDC device (with a maximal output voltage of 1400 V) to generate electric pulses with a voltage to distance ratio of 1000 V/cm. The electrodes and the ECT-SBDC device were designed in the electromagnetic laboratory of the Medical Physics department of Tarbiat Modares University, Tehran, Iran. Eight square-wave electric pulses (two trains of 4 pulses) of 1000 V/cm amplitude, with a pulse duration of 100μs and repetition frequency 1 Hz, were delivered by the two parallel electrodes which were placed on the skin at the opposite sides of the tumor. An optimal contact between the electrodes and the skin was assured by means of a conductive gel. In all the electrochemotherapy groups, mice were treated with the electric pulses 1 min after the cisplatin injection.

### Radiotherapy

A cobalt-60 unit (Theraton 780, Canada) was used to locally deliver a single dose of 3 Gy or 5 Gy at a dose rate of 0.6 Gy/min (3 Gy in 5 min) or 0.71 Gy/min (5 Gy in 7 min). Irradiation was performed frontally. As the smallest field covered by the Cobalt beam was 4 × 4 cm^2^, the mouse’s body around the tumor was shielded using lead blocks so as to leave only the tumor exposed to the irradiation beam.

### Study protocol

The experimental groups included: untreated tumors as controls, tumors treated with cisplatin, electric pulses or radiotherapy alone, tumors treated by electrochemotherapy (that is cisplatin injection followed by the local application of electroporative electric pulses) and tumors treated with the combination of cisplatin or electric pulses and radiotherapy. In the combination of cisplatin or electroporation with local radiotherapy, tumor-bearing mice were irradiated 30 min after the cisplatin injection or the electric pulses delivery, to provide the time to cisplatin to enter the cells.

### Treatment evaluation

Tumor growth was monitored, every three days, by measuring two mutually orthogonal tumor diameters (e1 being the larger tumor diameter and e2 the largest diameter orthogonal to e1). Tumors were rather spherical and tumor volumes were calculated by the formula V= π/6 × e1× e2^2^. From the tumor growth curves, tumor-doubling time (DT) was determined for each individual tumor. Tumor growth delay (GD) was calculated by subtracting the mean tumor volume doubling time of the untreated tumors (control) from the mean tumor volume doubling time of each experimental group. Inhibition ratio, expressed in percent, was calculated at day 30 after the treatment by the formula [1-(treated tumor average volume/untreated tumor average volume)] ×100%. Partial response (PR) was a decrease by more than 50% of the tumor volume. Complete response (CR) was the absence of tumor for more than 100 days.

Normalized volumes at day N for each animal were calculated by dividing the tumor volume Vn at day N after the treatment by the average tumor volume V0 at day 0 that is at the day of the treatment (Vn/V0).

### Statistical analysis

All data were tested for normality of distribution. The ANOVA test with repeated measures was used to evaluate statistical significance of differences between experimental and control groups at different times. A p-value<0.05 was considered significant in the statistical tests (*p <0.05).

## Results

**FIGURE 1:**
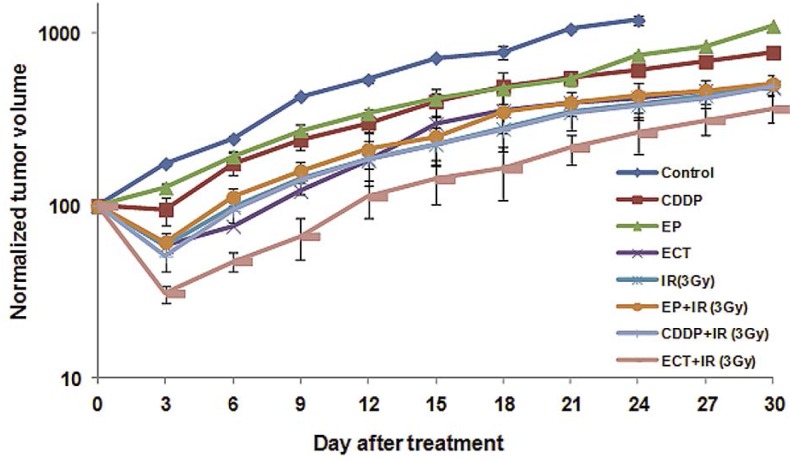
Tumor growth curves in untreated tumors or after treatment with CDDP (CDDP), electric pulses (EP) only, irradiation (IR) only, electrochemotherapy (ECT) and the combinations of CDDP or electric pulses and radiotherapy. Data are mean ± SE of at least 8 animals for each of the experimental groups. Irradiation of tumor was 3Gy.

As shown in Table 1 and Figure 1, cisplatin (at a dose of 8 mg/kg) as a single treatment delayed the tumor growth up to 5.5 days with an inhibition ratio of 33%. Tumors that were treated with electric pulses alone (8 pulses, 1000 V/cm, 100μs duration, at 1 Hz repetition frequency) displayed a tumor growth delay of only 2 days with an inhibition ratio of 32% (Table 1) and with no partial or complete responders with neither of both treatment modalities.

In the electrochemotherapy group, in which tumors were exposed to electric pulses 1 min after the cisplatin injection, a prolonged tumor growth delay (up to 15.5 days) was observed (p <0.05). The inhibition ratio reached 61% (Table 1). Thus, electrochemotherapy was more effective than either cisplatin alone or the application of electric pulses alone, with moreover 30% partial response for the first 9 days (p <0.05).

**FIGURE 2:**
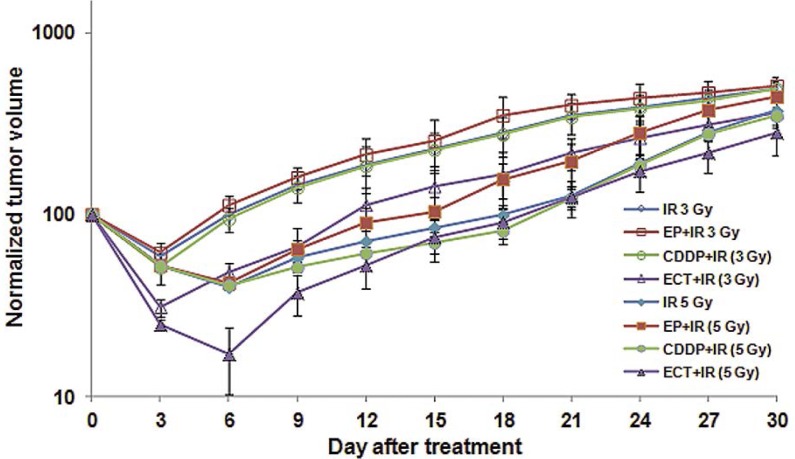
Tumor growth curves for invasive ductal carcinoma tumors after treatment with radiotherapy (IR) only, electrochemotherapy (ECT), combination of CDDP or electric pulses and radiotherapy at dose of 3 Gy in comparison to 5 Gy. Data are mean ± SE of at least 8 animals for each of the experimental groups.

With local irradiation alone (Table 1 and Figures 1 and 2), the higher dose (5 Gy) resulted in a growth delay up to 20.6 days. Tumors irradiated with a single dose of 3 Gy resulted in a tumor growth delay of 11.1 days and an inhibition ratio of 63% (Table 1). Some partial responses (25%) were observed within the 15 days that followed the administration of 5 Gy, but only 11% with 3 Gy.

When the administration of cisplatin was combined with local irradiation performed 30 min after the drug injection, the treatment of tumors resulted in tumor growth delays and inhibition ratios of 11.3 days and 61% respectively with a 3 Gy radiation and of 21.0 days and 75% with a 5 Gy irradiation (Table 1). These results demonstrate that a dose of 5 Gy was more effective. This is also borne out by the fact that 25% partial responses were observed for up to 15 days with the 3 Gy irradiation, whereas only 11% partial responses were recorded up to 12 days following the combination of cisplatin and 3 Gy irradiation (p <0.05).

When electric pulses were combined with local irradiation delivered 30 min after the electric pulses application, an improved antitumor effect was not observed compared to irradiation alone. Tumor growth delays of 8.8 days and inhibition ratio of 51% after 3 Gy and of 17.8 days and 59% after 5 Gy were scored (Table 1). With 5 Gy, one partial response (12.5%) was achieved for 15 days with no partial responders after 3 Gy.

**FIGURE 3:**
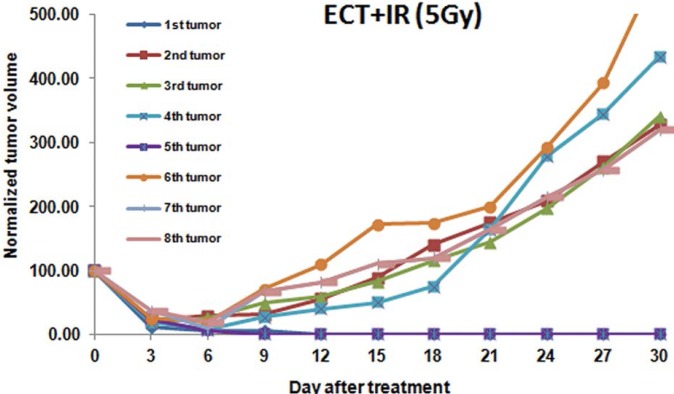
Individual tumor growth curves of tumors treated by the combination of electrochemotherapy (ECT) and radiotherapy at the dose of 5 Gy.

Irradiation of tumors pretreated with electrochemotherapy prolonged the tumor growth delay up to 25.7 days and 38.6 days at 3 Gy and 5 Gy respectively. The inhibition ratio reached 76% for the 3 Gy irradiation and 87% for 5 Gy. Ten per cent of partial responses after 30 days were observed with electrochemotherapy followed by 3 Gy, though without complete responders. In contrast, the electrochemotherapy with a 5 Gy irradiation resulted in a higher percentage of partial regressions (37% of the tumors were still in partial response 30 days after the treatment). Moreover, 25% of the mice were still in complete remission 100 days after the treatment (Table 1 and Figure 3). Again, the combination with 5 Gy was significantly (p <0.05) more efficient than the combination with 3 Gy. Even in this group, no body weight loss or skin desquamation was reported. Only a hair loss was observed in all the groups that were irradiated, locally at the level of the irradiation site.

In summary, in all the cases, the combination with γ- radiation resulted in a larger antitumor efficacy than without the combination with γ-rays.

## Discussion

In this study, the therapeutic effects of cisplatin, electroporation, γ-radiation and combination of these treatments were evaluated in a murine invasive ductal carcinoma tumor model. Our data confirm and extend previous findings^16^–^18^ showing that the delivery to tumors of electric pulses and cisplatin enhances the cisplatin-induced radiosensitization.

Electroporation combined with cisplatin has shown a significant effectiveness both *in vitro* and *in vivo,* as well as in clinical studies in the treatment of patients with cutaneous tumor nodules.^15^–^18^,^24^,^25^ Although the increased radiosensitizing effect of cisplatin using electroporation has been shown in EAT carcinoma and LPB sarcoma cells^27^,^28^, data on radiosensitization for large tumors, of a size closer to clinical situations, have not yet been reported. In this study, this issue was addressed and using local γ-radiation with single doses of 3 Gy or 5 Gy of Cobalt-60 γ-rays in combination with electrochemotherapy with cisplatin on large invasive ductal carcinoma tumors as a model for the treatment of large breast tumors.

Our study confirms that electrochemotherapy was more effective than individual treatments using cisplatin or electric pulses alone which is consistent with previous reports.^16^–^18^,^27^,^28^ When electrochemotherapy with cisplatin was combined with local irradiation, the antitumor efficiency was doubled. This three-modality therapy was better than electrochemotherapy, cisplatin combined with local tumor irradiation and electric pulses combined with irradiation. In addition, some tumors were still in complete remission 100 days after the treatment at single dose of 5 Gy. It is important to highlight that the irradiation doses used in the present study (a single 3 Gy or 5 Gy dose) are lower than the doses used in the previous experimental studies with cisplatin reported here above.^27^,^28^ Indeed, Sersa *et al*. used a dose of 15 Gy for electrochemotherapy with cisplatin combined with irradiation on the EAT tumor animal model.^27^ However, there are studies that demonstrated an increase of the tumor curability dose (TCD50) for both cisplatin (being 1.6) and bleomycin (1.9).^32^,^33^

This study concentrated on electrochemotherapy with cisplatin, as cisplatin is a radiosentizer, with the aim also to show that electrochemotherapy can radiosensitize the tumors. However, the initial goal was to determine whether electrochemotherapy efficacy could be increased by irradiation even in large tumors. Indeed, using about the same dose rate 2.2 Gy/min (10–20 Gy), Sersa *et al.* showed that efficacy of electrochemotherapy with bleomycin (a molecule considered a radiomimetic but not a radiosensitizer) was increased with irradiation, on SA-1 and CaNT tumor models.^32^

Finally it is important to report that our results are also in agreement with two clinical studies^34^,^35^ reporting that electrochemotherapy in combination with irradiation is an effective and safe treatment for tumors of various origins in patients, thus on lesions of a size at least comparable to those treated in this study.

In conclusion the results of this study demonstrate that electrochemotherapy with cisplatin radiosensitized the tumors to a single low irradiation dose (3 Gy or 5 Gy of Cobalt-60 γ-rays) on invasive ductal carcinoma tumors. Moreover, this three-modality combined treatment was useful for the treatment of large lesions even at sub-optimal doses of irradiation. Complete response of very large tumors could be achieved after a single session with the combined multimodality treatment.

## Figures and Tables

**TABLE 1:** Tumor growth after treatment with three modalities; cisplatin, electric pulses and local γ-radiation or combination on invasive ductal carcinoma tumor model

**Experimental groups**	**n**	**DT days, Mean±SE**	**GD days, mean**	**Inhibition ratio (%)**	**CR number**
**Control**	8	4.6±0.5		0	0
**CDDP**	8	10.6±1.21	5.5	33	0
**EP**	8	6.6±0.5	2.0	32	0
**ECT**	10	20.1±1.2	15.5	61	0
**IR (3 Gy)**	9	15.7±1.4	11.1	63	0
**CDDP + IR (3 Gy)**	9	15.9±2.4	11.3	61	0
**EP + IR (3 Gy)**	8	13.4±2.1	8.8	51	0
**ECT + IR (3 Gy)**	10	30.3±2.6	25.7	76	0
**IR (5 Gy)**	8	25.2±1.1	20.6	70	0
**CDDP + IR (5 Gy)**	8	25.6±1.2	21.0	75	0
**EP + IR (5 Gy)**	8	22.4±1.4	17.8	59	0
**ECT + IR (5 Gy)**	8	43.2±1.5	38.6	87	2

CDDP = cisplatin; EP = electric pulses; ECT = electrochemotherapy; IR = irradiation; n = number of mice in the experimental group; DT = tumor doubling time; GD = growth delay; PR = partial response; CR = complete response

## References

[1] Gately DP, Howell SB (1993). Cellular accumulation of the anticancer agent CDDP: a review. Br J Cancer.

[2] Kelland L (2007). The resurgence of platinum-based cancer chemotherapy. Nat Rev Cancer.

[3] Lekic M, Kovac V, Triller N, Knez L, Sadikov A, Cufer T (2012). Outcome of small cell lung cancer (SCLC) patients with brain metastases in a routine clinical setting. Radiol Oncol.

[4] Malecki K, Glinski B, Mucha-Malecka A, Rys J, Kruczak A, Roszkowski K (2010). Prognostic and predictive significance of p53, EGFr, Ki-67 in larynx preservation treatment. Rep Pract Oncol Radiother.

[5] Kovac V, Smrdel U (2004). Meta-analyses of clinical trials in patients with non-small cell lung cancer. Neoplasma.

[6] Erculj N, Kovac V, Hmeljak J, Dolzan V (2012). The influence of platinum pathway polymorphisms on the outcome in patients with malignant mesothelioma. Ann Oncol.

[7] Kelland LR (2000). Preclinical perspectives on platinum resistance. Drugs.

[8] Begg AC, Deurloo MJ, Kop W, Bartelink H (1994). Improvement of combined modality therapy with CDDP and radiation using intratumoral drug administration in murine tumors. Radiother Oncol.

[9] Sharma VM, Wilson WR (1999). Radiosensitization of advanced squamous cell carcinoma of the head and neck with CDDP during concomitant radiation therapy. Eur Arch Otorhinolaryngol.

[10] Bergs JWJ, Franken NAP, Cate RT, Bree CV, Haveman J (2006). Effects of CDDP and γ-irradiation on cell survival, the induction of chromosomal aberrations and apoptosis in SW-1573 cells. Mutat Res.

[11] Ning S, Yu N, Brown DM, Kanekal S, Knox SJ (1999). Radiosensitization by intratumoral administration of CDDP in a sustained-release drug delivery system. Radiother Oncol.

[12] Strojan P, Katarina K, Lojze S, Erika S, Igor F, Boris J, Aleksandar A, Marjan B, Branko Z (2008). Concomitant chemoradiotherapy with Mitomycin C and Cisplatin in advanced unresectable carcinoma of the head and neck: Phase I–II Clinical Study. Int J Radiat Oncol Biol Phys.

[13] Carde P, Laval F (1981). Effect of cis-dichlorodiammineplatinum (II) and X-rays on mammalian cell survival. Int J Radiat Oncol Biol Phys.

[14] Geldof AA, Slotman BJ (1996). Radiosensitizing effect of CDDP in prostate cancer cell lines. Cancer Lett.

[15] Melvik JE, Pettersen EO, Gordon PB, Seglen PO (1986). Increase in cis- dichlorodiammineplatinum (II) cytotoxicity upon reversible electro-permeabilization of the plasma membrane in cultured human NHIK 3025 cells. Eur J Cancer Clin Oncol.

[16] Cemazar M, Miklavcic D, Vodovnik L, Jarm T, Rudolf Z, Stabuc B (1995). Improved therapeutic effect of electrochemotherapy with cisplatin by intratumoral drug administration and changing of electrode orientation for electromeatabilization on EAT tumor model in mice. Radiol Oncol.

[17] Sersa G, Cemazar M, Miklavcic D (1995). Antitumor effectiveness of electrochemotherapy with cis-diamminedichloroplatinum (II) in mice. Cancer Res.

[18] Sersa G, Cemazar M, Semrov D, Miklavcic D (1996). Changing electrode orientation improves the efficacy of electrochemotherapy of solid tumors in mice. Bioelectrochem Bioenerg.

[19] Mir LM, Orlowski S, Jaroszeski MJ, Heller R, Gilbert R (2000). The basis of electrochemotherapy. Electrochemotherapy, Electrogentherapy, and Transdermal Drug Delivery Electrically Mediated Delivery of Molecules to Cells.

[20] Silve A, Mir LM, Kee ST, Gehl J, Lee EW (2011). Clinical aspects of electroporation. Cell electropermeabilization and cellular uptake of small molecules: the ECT concept.

[21] Mir LM, Orlowski S, Belehradek J, Paoletti C (1991). Electrochemotherapy potentiation of antitumor effect of bleomycin by local electric pulses. Eur J Cancer.

[22] Marty M, Sersa G, Garbay JR, Gehl J, Collins CG, Snoj M (2006). Electrochemotherapy—an easy, highly effective and safe treatment of cutaneous and subcutaneous metastases: results of ESOPE (European Standard Operating Procedures of Electrochemotherapy) study. EJC Suppl.

[23] Sersa G, Stabuc B, Cemazar M, Jancar B, Miklavcic D, Rudolf Z (1998). Electrochemotherapy with CDDP: potentiation of local CDDP antitumor effectiveness by application of electric pulses in cancer patients. Eur J Cancer.

[24] Cemazar M, Sersa G, Miklavcic D, Scancar J, Dolzan, Golouh R (1999). Increased platinum accumulation in SA-1 tumor cells after in vivo electrochemotherapy with CDDP. Br J Cancer.

[25] Sersa G, Stabuc B, Cemazar M, Miklavcic D, Rudolf Z (2000). Electrochemotherapy with cisplatin: clinical experience in malignant melanoma patients. Clin Cancer Res.

[26] Jarm T, Cemazar M, Miklavcic D, Sersa G (2010). Antivascular effects of electrochemotherapy: implications in treatment of bleeding metastases. Expert Rev Anticancer Ther.

[27] Sersa G, Kranjc S, Cemazar M (2000). Improvement of combined modality therapy with CDDP and radiation using electroporation of tumors. Int J Radiat Oncol Biol Phys.

[28] Kranjc S, Cemazar M, Grosel A, Scancar J, Sersa G (2003). Electroporation of LPB sarcoma cells *in vitro* and tumors *in vivo* increases the radiosensitizing effect of CDDP. Anticancer Res.

[29] Kranjc S, Cemazar M, Grosel A, Pipan Z, Sersa G (2003). Effect of electroporation on radiosensitization with cisplatin in two cell lines with different chemo- and radiosensitivity. Radio Oncol.

[30] Hassan ZM, Yaraee R, Chazanfari T, Safar Kejad AH, Nozari B (2003). Immunomodulatory affect of R10 fraction of garlic extract on natural killer activity. Int J Immunopharmaco.

[31] Raeisi E, Firoozabadi SMP, Hajizadeh S, Rajabi H, Hassan Z M (2010). The effect of high-frequency electric pulses on tumor blood flow in vivo. J Membrane Biol.

[32] Kranjc S, Tevz G, Vidic S, Cemazar M, Sersa G (2009). Radiosensitizing effect of electrochemotherapy in a fractionated radiation regimen in radiosensitive murine sarcoma and radioresistant adenocarcinoma tumor model. Radiat Res.

[33] Sersa G, Kranjc S, Cemazar M, Pakhomov A, Miklavcic D, Marko MS (2010). Advanced electroporation techniques in biology and medicine. Combined modality therapy: electrochemotherapy with tumor irradiation.

[34] Sersa G, Cemazar M, Rudolf Z, Fras AP (1999). Adenocarcinoma skin metastases treated by electrochemotherapy with cisplatin combined with radiation. Radiol Oncol.

[35] Skarlatos I, Kyrgias G, Mosa E, Provatopoulou X, Spyrou M, Theodorou K (2011). Electrochemotherapy in cancer patients: first clinical trial in Greece. In Vivo.

